# Predictors of prolonged length of stay in patients undergoing laser interstitial thermal therapy for intracranial tumors

**DOI:** 10.1007/s11060-025-05101-1

**Published:** 2025-06-16

**Authors:** Khushi H. Shah, Adham M. Khalafallah, Maxon V. Knott, Chandler N. Berke, Christian K. Ramsoomair, Victor M. Lu, Michael E. Ivan, Ricardo J. Komotar, Ashish H. Shah

**Affiliations:** 1 Department of Neurological Surgery, Miller School of Medicine, University of Miami, 1475 NW 12th Ave, Miami, FL 33136 USA; 2 Sylvester Comprehensive Cancer Center, University of Miami Health System, Miami, FL USA

**Keywords:** Hospital length of stay, Prolonged length of stay, Laser interstitial thermal therapy, Intracranial tumors

## Abstract

**Purpose:**

Laser interstitial thermal therapy (LITT) offers a minimally invasive approach for treating intracranial pathologies while offering shorter length of stays (LOS) as compared to traditional craniotomies. Yet, some patients still face prolonged LOS (pLOS), highlighting the need to identify factors contributing to pLOS to improve outcomes.

**Methods:**

We retrospectively reviewed patients who underwent LITT for intracranial pathologies at our institution from 2012 to 2023. Patients with LOS ≥ 75th percentile formed the study group, while those with LOS < 75th percentile formed control group. Patient demographics and perioperative factors were analyzed. Bivariate statistical analyses included Fisher’s exact test, chi-square test, and t-tests. Univariate and multivariate logistic regression identified significant predictors of pLOS.

**Results:**

Of 294 patients in this study, 73 patients in the study group (mean age 62.14 ± 11.63 years, 54.8% males) with a median LOS of 4.12 [IQR: 3.01–6.67] days were compared to 221 controls (mean age 59.50 ± 14.01 years, 40.3% males) with a median LOS of 1.92 [IQR: 1.86–2.01] days. Upon multivariate analysis, higher mFI-5 scores (OR 1.80; 95% CI [1.31–2.47]; *p* < 0.001), preoperative neurologic deficits (OR 2.27; 95% CI [1.09–4.76]; *p* = 0.029), and preoperative tumor volume (OR 2.03; 95% CI [1.46–2.83]; *p* < 0.001) were significantly associated with pLOS. Operative time, number of pullbacks, and extent of ablation were not significantly associated with pLOS (*p* > 0.05).

**Conclusion:**

To our knowledge, this is the first study to identify preoperative mFI-5 score, neurological deficit, and tumor volume as independent predictors of pLOS in patients undergoing LITT for intracranial pathologies.

## Introduction

Laser interstitial thermal therapy (LITT) has emerged as a minimally invasive, ablative technique for cytoreduction of intracranial pathologies [1]. It offers an alternative to traditional craniotomy, particularly in patients deemed unsuitable for surgery, with advantages including reduced length of stay (LOS), fewer postoperative complications, less pain, and decreased narcotic use [2–4]. In a multisite, prospective registry of LITT for abnormal neurological tissue, when both LITT and craniotomy were feasible, 58.6% of physicians stated that LITT was performed over craniotomy because the patient preferred a minimally invasive procedure [5].

Hospital LOS is a critical metric for evaluating care efficiency, with shorter stays reflecting effective care delivery, while prolonged stays (pLOS) are associated with increased resource utilization, higher healthcare costs, and elevated risk of patient complications [6–8]. LITT has been associated with more cost-effectiveness compared to craniotomy in the treatment of primary and metastatic brain tumors, radiation necrosis, and drug-resistant epilepsy [9–11]. Moreover, patients undergoing LITT had a mean LOS of 3.2 ± 0.3 days [12], as compared to a mean LOS of 6.55 ± 1.77 days for patients undergoing open craniotomy [13]. However, while most patients undergoing LITT experience short hospital stays, a subset experience pLOS. Therefore, it is important to identify predictors of pLOS to optimize care.

This study aims to identify clinical factors that contribute to pLOS following LITT for intracranial pathologies. By understanding how these factors influence LOS, this study seeks to identify patients at increased risk for prolonged hospitalization following LITT. This information will not only improve patient counseling regarding their expected hospital stay but also provide valuable insights for healthcare stakeholders. Ultimately, the goal is to enhance our understanding of factors influencing LOS after LITT, leading to more efficient patient care and better hospital resource management.

## Methods

### Patient selection

After Institutional Review Board Approval (IRB no. 20160437), we retrospectively review patients treated with LITT at our institution from February 2013 to August 2023. LITT was offered to patients deemed high-risk surgical candidates due to age, comorbidities, or tumor locations posing higher risks of postoperative neurological morbidity, as evaluated by primary neurosurgeons M.E.I., R.J.K., and A.H.S. Detailed information regarding our LITT protocol has been previously reported [14].

### Inclusion criteria

Patients with age ≥ 18 years who underwent LITT for intracranial tumors as confirmed by histopathological diagnosis with LOS data were included. Informed consent was waived due to the retrospective nature of this study. Given the absence of a standardized definition of pLOS in neurosurgical literature, we defined pLOS as LOS ≥ 75th percentile, a threshold commonly used in prior studies [7, 15, 16]. Patients with prolonged LOS as defined by ≥ 75th percentile of LOS were categorized under the study group, while rest comprised the control group.

### Data collection

Preoperative data such as patient demographics, preoperative deficits, Karnofsky performance score (KPS), modified 11-item frailty index (mFI-11), and modified 5-item frailty index (mFI-5) were collected. Tumor characteristics included newly diagnosed or recurrent tumor, location, laterality, and preoperative volume. Magnetic resonance imaging was reviewed, and the lesion volume was calculated as previously described [14]. Intraoperative data included operative time, ablation time, number of trajectories and pullbacks, and extent of ablation (EOA). EOA was calculated as the 24-hour post-operative ablation volume divided by the preoperative tumor volume and multiplied by 100. Data on outcomes included postoperative deficits, immediate postoperative complications, discharge disposition, and LOS. Immediate postoperative complications were defined as any complication occurring between the end of surgery and hospital discharge.

### Statistical analysis

Categorical variables were compared using chi-square and Fisher exact tests, as appropriate. Continuous variables were analyzed using either Student’s t-test or Welch’s t-test depending on the equality of variance tested via Levene’s test. Mean and standard deviation were reported for all continuous variables, except for those with non-normal distribution, where median and interquartile range (IQR: 25-75th percentile) were reported instead.

Univariate and multivariate logistic regression identified predictors of prolonged LOS. Continuous variables were standardized such that odds ratios reflected a one standard deviation increase in the variable. For categorical variables, odds ratios represented the effect of a one-unit increase in the variable’s interval. All variables significant upon univariate analysis, having adequate sample sizes (> 10 events), and no conceptual overlap were included in the multivariate model. For variables that measured the same underlying characteristic, the data with the most complete entries were selected for multivariate modeling to avoid collinearity issues. Between mFI-5 and mFI-11 scores, mFI-5 was selected for multivariate modeling given its simplified nature and can be easily integrated into clinical workflows [17]. Final variable selection included assumptions testing, correlation analysis, multicollinearity, and goodness of fit. Statistical significance was set at a p value < 0.05 for all analyses. Statistical analyses were performed using Python version 3.11.5 for MacOS and GraphPad Prism (version 10.1.2; GraphPad Software Inc.).

## Results

### Patient characteristics

**Fig. 1:**
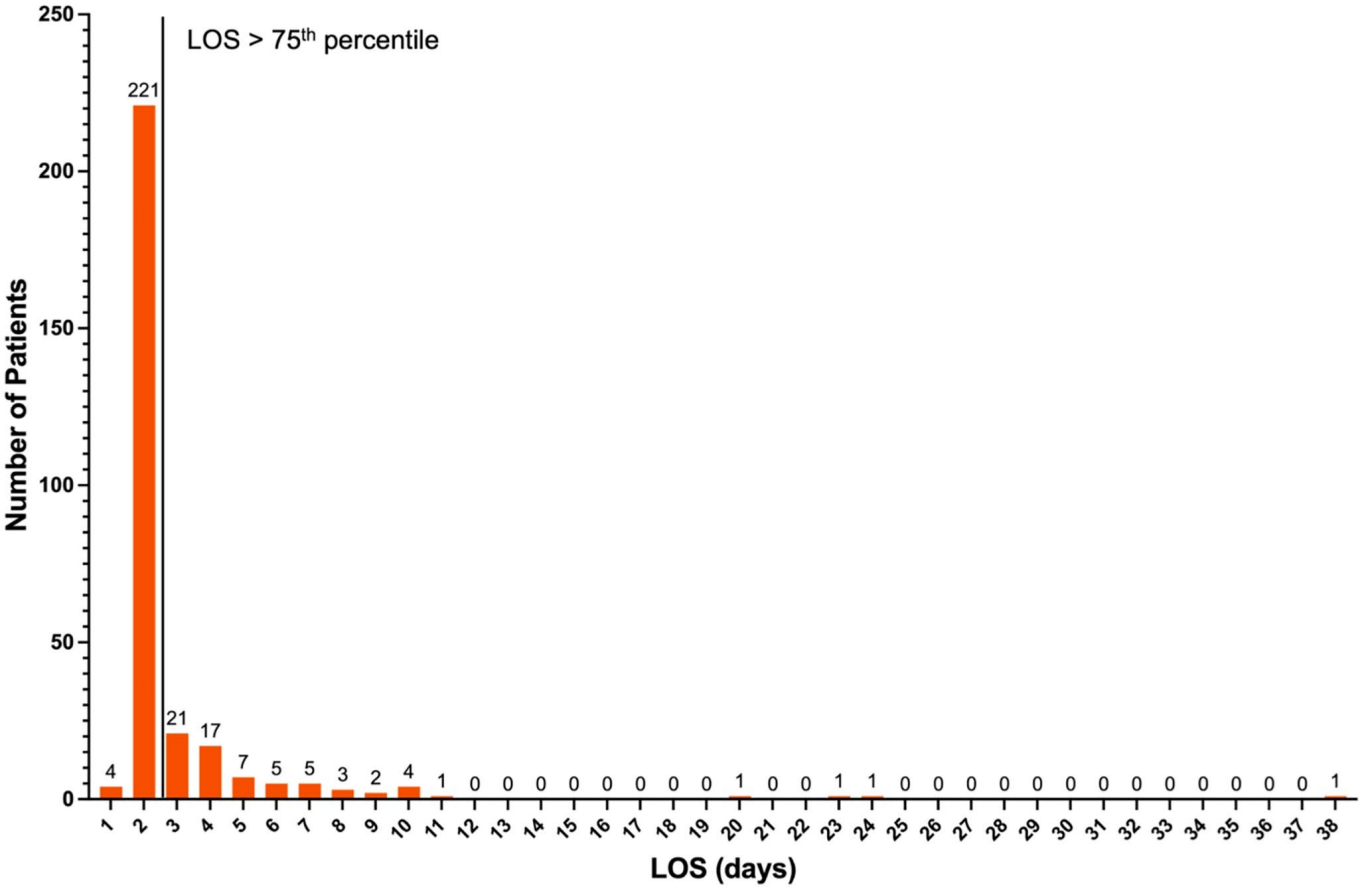
Histogram with LOS for overall cohort

During the study period, out of 313 patients who underwent the LITT at our institution, 294 patients met the inclusion criteria. This cohort had an average age of 60.15 ± 13.49 years, 43.58% male, and a median length of stay of 1.98 [IQR: 1.88–2.27] days (Fig. 1). The LOS was ≥ 75th percentile in 73 patients and they were categorized into the study group. The remaining 221 patients with LOS < 75th percentile comprised the control group. The median LOS in the study group was 4.12 days (range: 2.29–38.08 days) as compared to 1.92 days (range: 0.96–2.26 days) in the control group (*p* < 0.001).

Patient demographic and preoperative clinical and radiological characteristics are detailed in Table 1. There was no significant difference in age, race, ethnicity, and insurance between groups. The study group had a male preponderance (54.8% vs. 40.3%, *p* = 0.042) with greater rates of preoperative neurologic (84.9% vs. 63.5%, *p* = 0.001), motor (49.3% vs. 29.4%, *p* = 0.003), and speech deficits (12.3% vs. 4.5%, *p* = 0.038). The study group also had worse preoperative KPS (80 [IQR: 70–90] vs. 90 [IQR: 80–90], *p* < 0.001), higher mFI-5 score (2 [IQR: 1–2] vs. 1 [IQR: 0–1], *p* < 0.001), and higher mFI-11 score (2 [IQR: 1–3] vs. 1 [IQR: 0–2], *p* < 0.001) compared to the control group.

**Table 1:** Patient demographics and tumor characteristics

Variable	Study group, *n* = 73	Control group, *n* = 221	*P* value
**Patient demographics**			
Age (years), mean, SD	62.14 ± 11.63	59.50 ± 14.01	0.147
Gender, male	40 (54.8%)	89 (40.3%)	**0.042**
Race, non-white	8 (11.0%)	40 (18.1%)	0.126
Ethnicity, Hispanic	28 (39.4%)	57 (27.7%)	0.088
Insurance			0.087
Private	31 (42.5%)	103 (46.6%)	
Medicare	13 (17.8%)	17 (7.7%)	
Medicaid	27 (37.0%)	97 (43.9%)	
Uninsured	2 (2.7%)	4 (1.8%)	
Preop KPS, median, IQR	80 (70–90)	90 (80–90)	**< 0.001**
Preop mFI-5, median, IQR	2 (1–2)	1 (0–1)	**< 0.001**
Preop mFI-11, median, IQR	2 (1–3)	1 (0–2)	**< 0.001**
Preop neurologic deficit	62 (84.9%)	139 (63.5%)	**0.001**
Preop motor deficit	36 (49.3%)	65 (29.4%)	**0.003**
Preop speech deficit	9 (12.3%)	10 (4.5%)	**0.038**
Preop cognitive deficit	20 (27.4%)	56 (25.3%)	0.846
Preop seizure	12 (16.4%)	57 (26.0%)	0.131
**Tumor characteristics**			
Recurrent lesion	34 (46.6%)	139 (63.5%)	**0.016**
Preop tumor volume (cm^3^), median, IQR	8.64 (3.85–30.01)	3.76 (1.82–9.22)	**< 0.001**
Subcortical lesion	29 (41.4%)	63 (30.7%)	0.136
Infratentorial lesion	7 (9.6%)	20 (9.1%)	0.999
Side:			0.143
Right	31 (42.5%)	86 (39.3%)	
Left	35 (47.9%)	124 (56.6%)	
Bilateral	7 (9.6%)	9 (4.1%)	
Location:			**0.012**
Frontal	22 (30.1%)	80 (36.5%)	
Temporal	11 (15.1%)	61 (27.9%)	
Parietal	23 (31.5%)	37 (16.9%)	
Occipital	3 (4.1%)	13 (5.9%)	
Cerebellum	6 (8.2%)	19 (8.7%)	
Other	8 (11.0%)	9 (4.1%)	
Pathology			0.158
GBM	40 (54.8%)	85 (38.5%)	
HGG	0 (0.0%)	4 (1.8%)	
LGG	4 (5.5%)	22 (10.0%)	
Meningioma	0 (0.0%)	7 (3.2%)	
Metastasis	16 (21.9%)	63 (28.5%)	
Radiation necrosis	9 (12.3%)	26 (11.8%)	
Other	4 (5.5%)	14 (6.3%)	

Bold entries signify statistical significance, *p* < 0.05

KPS, Karnofsky performance scale; mFI-11, modified 11-item frailty index; preop, preoperative; GBM, glioblastoma; HGG, high grade glioma; LGG, low grade glioma

### Tumor characteristics

The study group had greater rates of newly-diagnosed lesions (53.4% vs. 36.5%, *p* = 0.016) and higher preoperative tumor volume (8.64 [IQR: 3.85–30.01] vs. 3.76 [IQR: 1.82–9.22] cm^3^, *p* < 0.001). While parietal tumor location was more common in the study group (31.5% vs. 16.9%, *p* = 0.012), there were no significant differences in the distribution of cortical versus subcortical lesions, supratentorial versus infratentorial lesions, hemispheric side, or pathology between the groups.

### Operative data

The mean operative time, ablation time, and number of pullbacks were greater in the study group, and the differences were statistically significant (Table 2). There was no significant difference between the number of trajectories between the two groups (*p* = 0.734). Moreover, EOA was significantly lower in the study group compared to the control group (122.35 [IQR: 100.58–160.00] vs. 137.38 [IQR: 105.98–214.00], *p* = 0.042).

**Table 2:** Operative characteristics

Variable	Study group, *n* = 73	Control group, *n* = 221	*P* value
Operative time (min)*, mean, SD	189.12 ± 70.13	158.75 ± 55.74	**0.001**
Ablation time (min)*, mean, SD	8.26 ± 4.46	7.19 ± 3.79	**0.070**
Trajectories			
1 trajectory	66 (93.0%)	209 (95.0%)	0.734
2 trajectories	5 (7.0%)	11 (5.0%)	
Number of pullbacks, mean, SD	3 (1–4)	2 (1–3)	**0.016**
EOA (%), mean, SD	122.35 (100.58–160.00)	137.38 (105.98–214.00)	**0.04** ***2***

*Data not available for all patients

Bold entries signify statistical significance, *p* < 0.05

EOA, extent of ablation

### Outcomes

There was no significant difference in new onset speech deficits between groups (Table 3). However, the study group had higher rates of immediate postoperative complications (23.3% vs. 0.9%, *p* < 0.001), new onset motor deficit (4.1% vs. 0.0%, *p* = 0.015), new onset postoperative seizures (4.1% vs. 0.0%, *p* = 0.015), and non-home discharge (8.3% vs. 0.5%; *p* = 0.001).

**Table 3:** Treatment outcomes

Variable	Study group, *n* = 73	Control group, *n* = 221	*P* value
LOS (days), median, IQR	4.12 (3.01–6.67)	1.92 (1.86–2.01)	**< 0.001**
New postop motor deficit	3 (4.1%)	0 (0.0%)	**0.015**
New postop speech deficit	1 (1.4%)	1 (0.5%)	0.436
New postop seizures	3 (4.1%)	0 (0.0%)	**0.015**
Patients with postoperative complications	17 (23.3%)	2 (0.9%)	**< 0.001**
Total number of complications	31	2	
Neurosurgical complications	8 (25.8%)	0 (0%)	0.999
Non-neurosurgical complications	23 (74.2%)	2 (100%)	0.999
Non-home discharge disposition	6 (8.3%)	1 (0.5%)	**0.001**

*Data not available for all patients

Bold entries signify statistical significance, *p* < 0.05

LOS, length of stay; postop, postoperative

Overall, 19 (6.5%) patients in our cohort experienced 33 complications during their initial hospital stay. In the study group, 17 (23.3%) patients experienced 31 complications, of which 8 were neurosurgical complications. Neurosurgical complications included seizure (*n* = 3), dysarthria (*n* = 1), dysphagia requiring NGT insertion and frequent suctioning (*n* = 1), hydrocephalus requiring VP shunt placement (*n* = 1), CSF leakage from burr hole site concerning for hydrocephalus managed with large-volume lumbar puncture and Diamox (*n* = 1), and meningitis (*n* = 1). Of the 2 patients with CSF-related complications, 1 had a periventricular lesion while the other had a lesion in the left frontal lobe. Non-neurosurgical complications were as follows: acute hypoxemic respiratory failure (*n* = 2), acute kidney failure with tubular necrosis (*n* = 1), atrial fibrillation (*n* = 2), anaphylactic cardiac arrest (*n* = 1), bradycardia (*n* = 2), death secondary to acute hypoxemic respiratory failure (*n* = 1), deep venous thrombosis (*n* = 1), hyperglycemia (*n* = 2), hypertension requiring cardiology follow up (*n* = 1), hyponatremia (*n* = 1), pneumonia (*n* = 3), severe osteopenia with multiple compression fracture needing thoracic vertebroplasty (*n* = 1), steroid induced Cushing syndrome (*n* = 1), superficial venous thrombosis (*n* = 1), and urinary retention (*n* = 3).

In contrast, 2 patients experienced immediate postoperative complications in the control group, which included 0 neurosurgical complications and 2 non-neurosurgical complications. Non-neurosurgical complications included hypertension requiring cardene drip overnight (*n* = 1) and acute kidney injury resolved with IV fluids (*n* = 1).

### Univariate and multivariate logistic regression

Upon univariate analysis, male gender, higher mFI-5, mFI-11, preoperative neurological deficits, preoperative motor deficit, preoperative speech deficit, preoperative tumor volume, tumor location, operative time, and number of pullbacks were associated with increased odds of pLOS (*p* < 0.05) (Table 4). In contrast, higher preoperative KPS, recurrent lesions, and higher EOA were associated with decreased odds of pLOS (*p* < 0.05).

**Table 4:** Univariate and multivariate logistic regression analysis of variables affecting LOS

Covariates	Univariate analysis				Multivariate analysis				
	OR	OR lower 95%	OR upper 95%	*P* value	OR	OR lower 95%	OR upper 95%	*P* value	VIF
Gender, male	1.80	1.05	3.06	**0.031**	1.39	0.67	2.86	0.372	1.09
Preop KPS	0.94	0.92	0.96	**< 0.001**					
Preop mFI-5	1.80	1.37	2.37	**< 0.001**	1.91	1.30	2.80	**0.001**	1.06
Preop mFI-11	1.77	1.36	2.32	**< 0.001**					
Preop neurologic deficit	3.24	1.61	6.52	**0.001**	3.17	1.23	8.15	**0.017**	1.09
Preop motor deficit	2.34	1.36	4.02	**0.002**					
Preop speech deficit	2.97	1.16	7.62	**0.024**					
Recurrent tumor	0.50	0.29	0.86	**0.012**	0.93	0.44	1.97	0.844	1.16
Preop tumor volume	2.09	1.54	2.83	**< 0.001**	1.61	1.06	2.43	**0.026**	1.45
Tumor location	1.20	1.01	1.42	**0.035**					
Operative time	1.64	1.20	2.24	**0.002**	1.47	1.00	2.16	0.052	1.10
Ablation time	1.07	0.99	1.15	0.073					
Number of pullbacks	1.27	1.05	1.52	**0.012**	1.07	0.81	1.41	0.633	1.34
EOA	0.38	0.19	0.79	**0.009**	0.69	0.30	1.58	0.375	1.15

Bold entries signify statistical significance, *p* < 0.05

KPS, karnofsky performance scale; mFI-5, modified 5-item frailty index; mFI-11, modified 11-item frailty index; preop, preoperative; EOA, extent of ablation; postop, postoperative

**Fig. 2:**
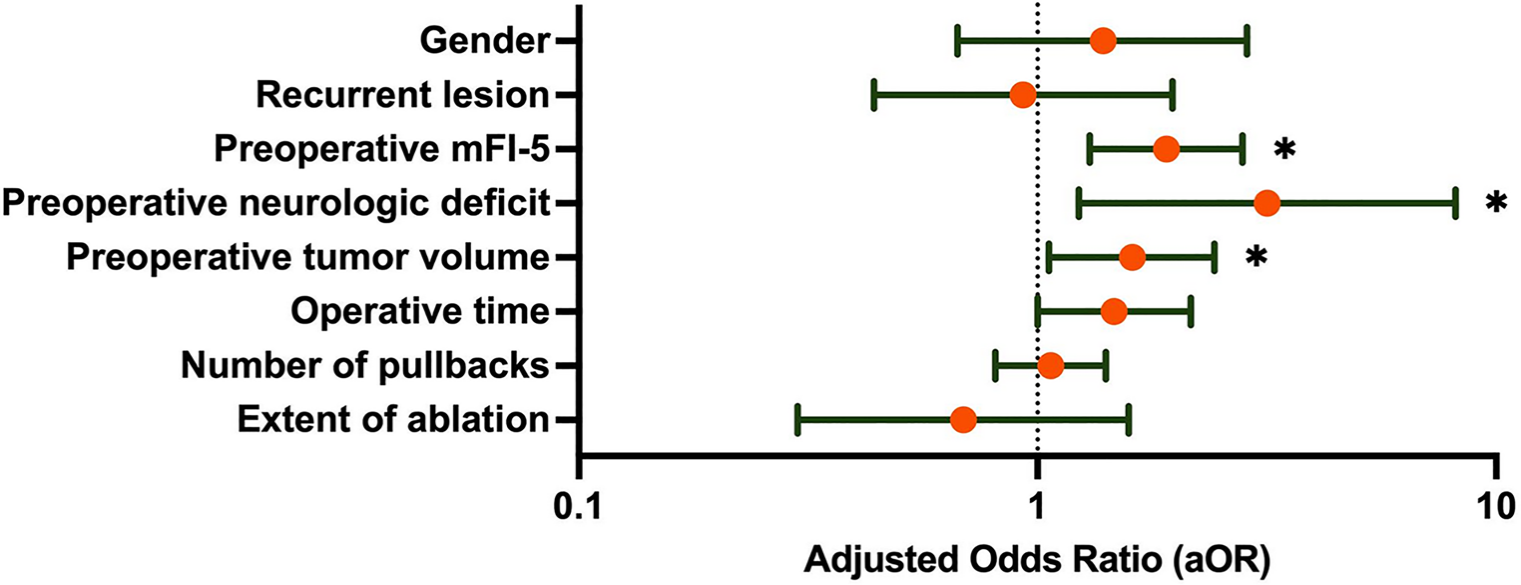
Forest plot with multivariate logistic regression of variables associated with pLOS in patients undergoing LITT. *p < 0.05

Upon multivariable regression, preoperative mFI-5 (OR 1.80; 95% CI [1.31–2.47]; *p* < 0.001), preoperative neurologic deficit (OR 2.27; 95% CI [1.09–4.76]; *p* = 0.029), and preoperative tumor volume (OR 1.99; 95% CI [1.45–2.73]; *p* < 0.001) were independently associated with pLOS (Fig. 2). In contrast, EOA (*p* = 0.375), number of pullbacks (*p* = 0.633), and operative time (*p* = 0.052) were not significantly associated with pLOS.

Given that preoperative mFI-5, neurologic deficits, and tumor volume were independently associated with pLOS on multivariate analysis, we further evaluated their association with immediate postoperative complications. On univariate logistic regression, mFI-5 was not significantly associated with postoperative complications (OR 0.94; 95% CI [0.59–1.51]; *p* = 0.805). In contrast, both preoperative neurologic deficits (OR 8.85; 95% CI [1.61–67.36]; *p* = 0.035) and preoperative tumor volume (OR 1.42; 95% CI [1.03–1.94]; *p* = 0.031) were significantly associated with increased odds of postoperative complications.

## Discussion

Many studies have shown LITT for intracranial pathologies to have a shorter LOS compared to craniotomies [10, 18, 19]. Moreover, in a cost analysis study comparing LITT and craniotomy, Leuthardt et al. concluded that, in addition to having a significantly shorter LOS (2.33 days vs. 4.71 days; *p* < 0.001), LITT was less costly than craniotomy for metastatic brain tumors [10]. LITT has also been associated with greater cost-effectiveness compared to craniotomy in the treatment of radiation necrosis and drug-resistant epilepsy [9, 11]. Overall, LITT’s cost-effectiveness and shorter LOS associated with LITT make it a reasonable option for bundled payment for care improvement (BCPI) programs for patients undergoing treatments for intracranial pathologies, particularly in deep-seated locations.

The BPCI program aims to administer value-based care to reduce costs by incentivizing care coordination and improved quality [20]. This program incentivizes hospital leadership to provide high-quality patient care while reducing excessive utilization of hospital resources [21, 22]. This includes reducing pLOS, which can lead to a misallocation of hospital resources and an increased risk of patient complications [7, 8]. Multiple studies show LITT to be associated with a short LOS with a median LOS of 2–3 days [23–25]. However, despite this short LOS associated with LITT, a subset of patients does experience pLOS. To the best of our knowledge, our study is the first to evaluate various factors that might be associated with pLOS after LITT.

### Study overview

In our study, upon multivariate regression, preoperative mFI-5, neurological deficit, and preoperative tumor volume were independently associated with pLOS. While a higher mFI-5 was not associated with increased risk of postoperative complications, tumor volume and neurological deficits were, suggesting different mechanisms by which these factors contribute to pLOS.

### Frailty

In our study, frailty, as characterized by preoperative mFI-5 score, was independently associated with pLOS in patients undergoing LITT for intracranial pathologies. We did not encounter any study evaluating the impact of frailty on LOS in patients undergoing LITT. However, there are abundance of studies on the impact of frailty on LOS in patients undergoing craniotomy for tumor resection [26–32]. Data from the Nationwide Readmissions database with 87,835 patients undergoing craniotomy for brain tumor noted frailty to be an independent predictor of in-hospital complications, non-routine discharge, and prolonged LOS [28]. A meta-analysis incorporating 52 publications encompassing 294,373 patients showed frailty to be associated extended LOS across most brain tumor studies [33]. Moreover, a large multicenter study analyzing the impact of frailty on complications and poor discharge outcomes noted that frailty, as characterized by mFI-5 and RAI-A, was significantly associated with extended LOS [31]. Specifically, a single center study on patients undergoing craniotomy characterized each 1-point increase in mFI-5 to be associated with a 1.38 day increase in LOS [32].

### Preoperative neurologic deficit

While no studies have analyzed the impact of preoperative neurologic deficits on pLOS in LITT, studies on open craniotomy have shown it to be a significant factor [34, 35]. Our findings are similar to a national database study on patients undergoing craniotomy for brain tumors which revealed the presence of impaired sensorium and hemiplegia preoperatively to be significant predictors of pLOS [34]. Similarly, another national database study revealed LOS was most strongly influenced by the presence of preoperative paralysis and non-paralysis neurological deficits, which independently increased the risk of extended LOS in craniotomy patients by 20.4% and 38.3% respectively [35]. In our study, we found a similar trend of preoperative neurologic deficits being independently associated with greater likelihood of pLOS. These findings suggest that even within the context of a minimally invasive approach such as LITT, baseline functional limitations still play a critical role in predicting hospital resource utilization. While LITT may offer faster recovery and reduced morbidity compared to open craniotomy, patients with significant neurologic impairments may still require longer hospitalization to address their complex needs.

### Preoperative tumor volume

Our study noted that a one standard deviation increase in preoperative tumor volume was statistically significantly associated with pLOS. This is in contrast with other studies in neurosurgical literature on LITT that indicate that preoperative tumor volume is not associated with increased LOS [36, 37]. A meta-analysis reviewing outcomes of LITT for recurrent glioblastomas showed studies with a consistent mean LOS of 1–2 days despite varying mean cohort tumor volumes [38]. Moreover, while evaluating the outcomes of LITT for glioblastoma for tumor sizes < 10 cm^3^ and ≥ 10 cm^3^, a study noted no significant difference in LOS between groups (*p* = 0.494) [36]. Lastly, a study by O’Conner et al. found that need of additional fibers for increasing tumor volume did not negatively impact LOS [37]. Contrary to these findings, in present study, there was a statistically significant difference in preoperative tumor volume between the two groups (8.64 cm^3^ in study group vs. 3.76 cm^3^ in control group). The reason for this discrepancy remains unclear. It is possible that differences in imaging acquisition, factors such as peritumoral edema, mass effect, or tumor proximity to eloquent structures could influence recovery trajectories. Given these potential contributors, further studies are warranted to clarify the impact of tumor volume on LOS following LITT.

### Impact of modifiable variables on pLOS

While operative time, number of pullbacks, and EOA were associated with pLOS on univariate analysis, none remained significant in multivariate modeling, indicating that intraoperative complexity alone does not independently prolong hospitalization. Instead, patient-level factors such as frailty, tumor volume, and neurologic deficits played a stronger predictive role. This distinction is important when counseling patients and designing perioperative protocols, as it underscores the need to prioritize early identification and optimization of at-risk patients rather than procedural refinements alone.

### Further considerations for pLOS in LITT

It is worth considering whether the extended LOS observed in frail patients undergoing LITT reflects increased vulnerability to LITT-specific complications or simply a need for prolonged hospitalization due to baseline limitations. Frailty, as characterized by mFI-5 scores, was independently associated with prolonged hospitalization but not with postoperative complications. In contrast, larger preoperative tumor volume and preexisting neurologic deficits were both significantly associated with higher complication rates. This suggests that frail patients may have an intrinsically diminished physiologic reserve, rather than increased perioperative risk. Frailty may prolong hospitalization due to functional challenges rather than procedural risk.

This is in comparison to tumor burden and neurologic status which more directly influence complications. While tumor volume and neurologic deficits are interrelated– larger lesions may be more likely to cause preoperative deficits– our collinearity analysis confirmed that both retained independent predictive value in multivariate modeling. This indicates that each factor may contribute to prolonged LOS through distinct but overlapping pathways.

Recognizing this distinction is critical for surgical planning and preoperative counseling. Frail patients may require enhanced discharge planning and rehabilitation support, whereas those with substantial tumor burden or neurologic symptoms may benefit from closer intraoperative monitoring and postoperative surveillance. Stratifying patients by these factors may guide more personalized care and optimize outcomes following LITT.

### Limitations and strengths

Our study is inherently limited by its retrospective nature and potential selection bias. To overcome this limitation, we included only patients with complete records available. Additionally, it is a single-institution study, but it involved patients managed under three neurosurgeons. Future studies and validation in larger cohorts and multi-institutional collaborations are crucial to validate our findings.

We defined pLOS using the 75th percentile, consistent with prior literature, though this threshold is statistically convenient rather than clinically grounded [7, 15, 16]. As pLOS lacks a standardized definition across studies– ranging from median-based to absolute day or percentile cutoffs– identified predictors may vary by definition [39].

Additionally, we did not collect data on preoperative or postoperative steroid use and therefore could not assess if steroid use could impact LOS. Moreover, we did not collect radiographic factors such as postoperative mass effect, midline shift, and edema extent and therefore, the correlation of these variables with pLOS could not be analyzed.

Despite these limitations, this is the first study to evaluate factors associated with pLOS in patients undergoing LITT for intracranial pathologies. By identifying factors linked to pLOS, our study aims to assist physicians and hospitals in proactively recognizing patients at higher risk for extended hospital stays, thereby promoting more efficient healthcare delivery. Optimizing costs and hospital resource allocation will not only enable hospitals to benefit from BPCI models but also expand access to LITT for more patients, improving overall care equity and availability.

## Conclusion

To the best of our knowledge, our study is the first to assess predictors of pLOS in patients undergoing LITT for intracranial tumors. We found preoperative mFI-5 score, neurologic deficits, and tumor volume to be factors independently associated with pLOS.

## Data Availability

No datasets were generated or analysed during the current study.
